# The Foreign Journals: Anatomy and Physiology

**Published:** 1840-01

**Authors:** 


### PART THIRD.

### Selections from the British and Foreign Journals.

### I. THE FOREIGN JOURNALS.

### ANATOMY AND PHYSIOLOGY.

*Further Experiments in the Inoculation of Animals with the Blood of Cholera Patients.* By SIGNOR NOVATI.

IN our Ninth Number we gave an account of a series of experiments performed by Dr. Namias, of Venice, upon dogs and rabbits with the blood of cholera patients, in almost all of which the disease appeared to be communicated, and was followed by the death of the animals. These experiments have been repeated by Dr. Campelio Calderini, of Milan, with nearly similar results; by Drs. Borsani, Freschi, and Novati, of Bergamo, without producing any injury to the animals; and upon four rabbits, four guinea-pigs, and two dogs, by Dr. Semola, of Naples, with no greater effect; and a commission of enquiry was instituted by the Royal Academy of Sciences to repeat these experiments, and report upon them. This was done upon eleven animals, rabbits, guinea-pigs, and dogs, and the result having been the same, the original Memoir of Semola has been inserted in the acts of the academy, and in the civil annals of the kingdom. Previous to this, however, Dr. Novati, not being satisfied with the first series of his experiments, resolved to repeat them upon a larger scale. The following are the results:

Two inoculations performed upon rabbits with the blood of living cholera patients produced no effects. Of twenty-one rabbits inoculated with human blood taken after death, eleven died between three and seven days afterwards, two of them having been inoculated with the blood of a fœtus of eight months, whose mother was affected with the disease; and the greater part of the survivors exhibited symptoms that, if not pathognomonic, at least are always observed in cholera. Of twenty-one other rabbits inoculated with the blood of one of the subjects of the former experiment, nineteen died between the first and fourth day, and the two others suffered in the same manner as the survivors from that experiment; thus proving, as had been before shown by Dr. Namias, that transmission through several individuals does not weaken the effect of the poison; and that it is more powerful when communicated from one animal to another of the same, than of a different class. The blood of a living cholera patient was injected into the mouth of one rabbit, and into the rectum of another, but neither of them experienced any ill effects. Another made to swallow some blood from a corpse, did not suffer more than it might have done from the mere operation.

Blood from a patient and a corpse was injected into the veins of two rabbits, the former died upon the spot, the latter in thirty hours; and the blood of the former rabbit injected into eight others destroyed seven of them very speedily. Another, whose femoral vein had been tied, was inoculated on the inside of the thigh without injury.

Fluid human dejections were inoculated with a vaccination needle, without effect; but those of a rabbit, inserted into incisions in the skin of three others, destroyed two, one in twelve hours, the other on the third day, the third rabbit escaping with difficulty.

A large rabbit was enveloped in the shirt of a cholera patient imbued with perspiration, but, except extreme prostration, the animal suffered no bad effects. Guided by the analogy of the effects of arsenic and the cholera, Novati mingled the tritoxide of iron, the newly-discovered remedy against this poison, with cholera blood, and injected it, when it caused death in nineteen hours. Arsenic itself produced death in a still shorter time. Common salt mingled with cholera blood was fatal in sixteen hours.

Besides these experiments, Sig. Novati performed many others upon rabbits, by wounding them severely, and injecting the blood of persons affected with various diseases, but without ever being able to produce similar effects. We have only given the results, but every minute circumstance connected with the experiments is noted in the original memoir.

*Annali Universali di Medicina,* 1838. Vol. lxxxv.

### *Petrifaction of the Tissues.*

The account which was given in our Number for Oct. 1836, of the conversion of the tissues of the human body into a substance of a strong hardness, by a mode of preparation discovered by Signor Segato, of Florence, is fully confirmed by M. Moreau, in the *Journal des Connaissances Medico-Chirurgicales.* Segato died three years since, without having revealed the secret of his art; but a large collection of his works is still exhibited in Florence. It consists of a great number of entire and variously dissected animals, fish, reptiles, birds, and small quadrupeds; of different organs, as the liver, spleen, placenta, brain, heart, &c.; of dissected limbs, clots of blood, &c. All the animal substances are converted into a material so hard that it can scarcely be filed, but will receive as fine a polish as marble, or any hard stone. Their colour, form, and size are unaltered; the minute vessels can be traced in them; and, but for their hardness, they might be taken for the real parts recently removed from the body.

*Journ. des Conn. Med.-Chir., Avril,* 1839.

[Signor Segato's process is not new, though he has employed it to a greater extent and with more success than Ruysch, to whom the same or a similar mode of preparation was well known. In his answer to the second letter of Gaubius, (*De artificiosa Scroti Humani induratione.* Sc. Op. omn. vol. i.,) Ruysch says, "You ask me to tell you of the preparation in which the scrotum is hardened like a stone. That is done by an artifice so singular that I laboured at it for more than four and thirty years. I hold nothing so difficult as to prepare parts of the human body, and especially the scrotum, so that their natural colour and size being preserved, and no unnatural wrinkles being formed, they should be hardened like stone, and yet that the course of their vessels should be far more distinct than in a living or in a recently dead body. But I cannot yet comply with your wish, nor can I be induced to make that preparation publicly known; and I am sure you will not require it of me when you consider with me how many there are who, like the Æsopian crow, delight to boast in others' plumes."]

